# Preoperative three-dimensional simulation for maxillary anterior bone augmentation with sagittal split ramus osteotomy: a case report

**DOI:** 10.1093/jscr/rjaf188

**Published:** 2025-04-15

**Authors:** Taifu Hirano, Tadashi Kawai, Yunosuke Ikeda, Shinsuke Kawamata, Shintaro Kogi, Mitsuru Izumisawa, Akihiro Fukutoku, Kazuhiro Kon, Hiroyuki Yamada

**Affiliations:** Division of Oral and Maxillofacial Surgery, Department of Reconstructive Oral and Maxillofacial Surgery, School of Dentistry, Iwate Medical University, 19-1 Uchimaru, Morioka, Iwate 020-8505, Japan; Division of Oral and Maxillofacial Surgery, Department of Reconstructive Oral and Maxillofacial Surgery, School of Dentistry, Iwate Medical University, 19-1 Uchimaru, Morioka, Iwate 020-8505, Japan; Division of Oral and Maxillofacial Surgery, Department of Reconstructive Oral and Maxillofacial Surgery, School of Dentistry, Iwate Medical University, 19-1 Uchimaru, Morioka, Iwate 020-8505, Japan; Division of Oral and Maxillofacial Surgery, Department of Reconstructive Oral and Maxillofacial Surgery, School of Dentistry, Iwate Medical University, 19-1 Uchimaru, Morioka, Iwate 020-8505, Japan; Division of Oral and Maxillofacial Surgery, Department of Reconstructive Oral and Maxillofacial Surgery, School of Dentistry, Iwate Medical University, 19-1 Uchimaru, Morioka, Iwate 020-8505, Japan; Division of Dental Radiology, Department of General Dentistry, Iwate Medical University, 19-1 Uchimaru, Morioka, Iwate 020-8505, Japan; Division of Fixed Prosthodontics and Oral Implantology, Department of Prosthodontics, School of Dentistry, Iwate Medical University, 19-1 Uchimaru, Morioka, Iwate 020-8505, Japan; Division of Fixed Prosthodontics and Oral Implantology, Department of Prosthodontics, School of Dentistry, Iwate Medical University, 19-1 Uchimaru, Morioka, Iwate 020-8505, Japan; Division of Oral and Maxillofacial Surgery, Department of Reconstructive Oral and Maxillofacial Surgery, School of Dentistry, Iwate Medical University, 19-1 Uchimaru, Morioka, Iwate 020-8505, Japan

**Keywords:** particulate cancellous bone and marrow, sagittal split ramus osteotomy, three-dimensional simulation, custom-made titanium mesh

## Abstract

Alveolar bone defects can result from trauma, resection of benign or malignant tumors, congenital anomalies, or periodontitis. These defects require bone augmentation to restore normal occlusal function and improve esthetics. This case report describes a 26-year-old man referred to our hospital with a diagnosis of jaw deformity. A deficiency of alveolar bone and teeth in the maxillary anterior region, caused by childhood trauma, was confirmed. Sagittal split ramus osteotomy was performed, accompanied by bone augmentation of the maxillary anterior region using a custom-made titanium mesh and autogenous iliac particulate cancellous bone and marrow, guided by three-dimensional simulation. Dental implants were placed in the augmented region 4 months later, followed by prosthetic treatment. This case demonstrates successful esthetic and functional reconstruction of a maxillary anterior bone defect and jaw deformity using preoperative three-dimensional simulation.

## Introduction

Autologous bone grafting has long been a standard method for reconstructing extensive jawbone defects [1, 2]. Recently, titanium mesh trays combined with autogenous iliac particulate cancellous bone and marrow (PCBM) have emerged as effective treatments for such defects [3, 4]. PCBM was first introduced as a grafting material by Mowlem in 1944 [5]. However, when bone augmentation and dental implant placement are needed in the maxillary anterior region, performing sagittal split ramus osteotomy (SSRO) can be challenging because of the difficulty in predicting occlusion. We herein present a case of skeletal mandibular protrusion with loss of alveolar bone and teeth in the maxillary anterior region. Surgery was planned using a three-dimensional (3D) model, enabling simultaneous SSRO and bone augmentation using a titanium mesh and PCBM. This is the first report of this combined approach for a maxillary anterior alveolar defect. We provide an overview of the case and preoperative plan.

## Case report

**Figure 1:**
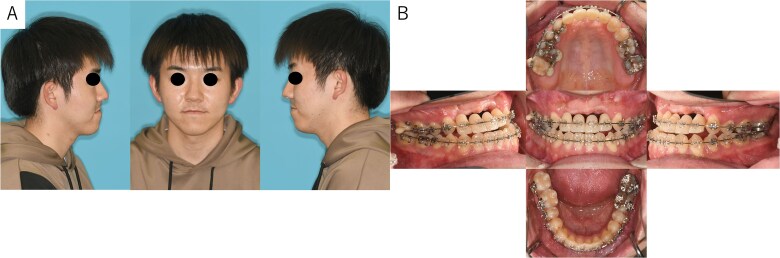
Patient photographs. (A) Extraoral photograph showing symmetrical facial features with a straight lateral profile and mandibular protrusion. (B) Intraoral examination revealed alveolar bone defects in the anterior maxillary teeth region. Temporary anterior teeth were present, with an overbite of +1 mm and an overjet of −7.3 mm. The angle classification of malocclusion was class I.

**Figure 2:**
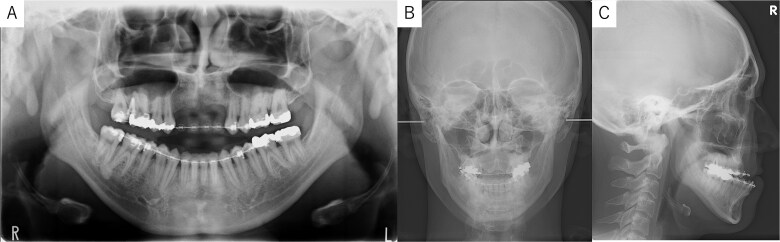
Panoramic radiograph and cephalogram. (A) Panoramic radiograph showing bone loss in the maxillary anterior region from the right to left canine. (B) Frontal cephalogram showing no chin deviation. (C) Lateral cephalogram indicating skeletal mandibular protrusion.

A 26-year-old man was referred to our department in February 2023 for surgical orthodontic treatment and bone grafting. He had experienced tooth loss and an alveolar bone defect due to trauma at the age of 2 years. The patient was 174.5 cm tall, weighed 69.7 kg, and showed normal nutritional and blood test results. His facial features were symmetrical, with a straight lateral profile and mandibular protrusion (Fig. 1A). Intraoral examination revealed missing bilateral maxillary central incisors, lateral incisors, and canines, along with an anterior maxillary alveolar bone defect. Temporary anterior teeth were present, with an overbite of +1 mm and an overjet of −7.3 mm. The maxillary and mandibular midlines aligned with the facial midline (Fig. 1B). A panoramic radiograph confirmed the tooth loss and alveolar defect (Fig. 2A), while cephalometric radiographs showed no chin deviation (Fig. 2B and C). Lateral cephalometric analysis (Table 1) revealed that the maxilla aligned with the anterior cranial floor and that the mandible had a high angle, consistent with skeletal mandibular protrusion. The clinical diagnosis was an alveolar bone defect in the maxillary anterior region and skeletal mandibular protrusion with a dolichofacial pattern.

**Table 1:** Cephalometric analysis. Lateral cephalometric analysis revealed that the maxilla aligned with the anterior cranial floor and that the mandible had a high angle, consistent with skeletal mandibular protrusion.

Measurement	Mean ± SD	Case
Facial angle	85.1 ± 5.8	82.7
Y-axis	65.7 ± 3.3	68.4
SNA	81.8 ± 3.1	78.1
SNB	78.6 ± 3.1	79.7
ANB	3.3 ± 2.7	−1.6 ↓
FMA	26.3 ± 6.3	39.2 ↑
Gonial angle	119.4 ± 5.8	136.0 ↑
Interincisal angle	129.7 ± 9.0	134.4
U1 to FH	108.9 ± 5.6	103.3
U1 to SN	103.1 ± 5.5	99.3
L1 to Mandibular plane	94.7 ± 7.2	83.0 ↓

**Figure 3:**
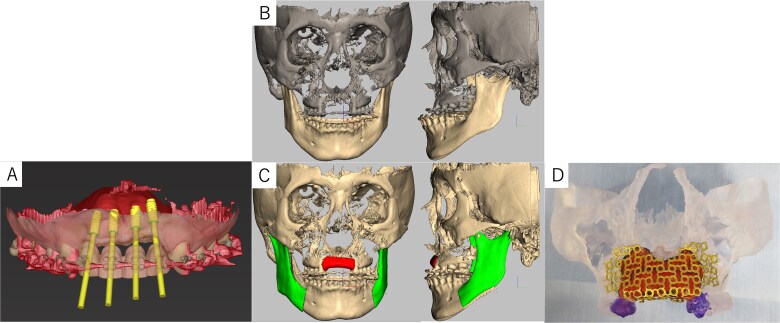
Virtual maxilla reconstruction and SSRO procedures using computed tomography data. (A) Simulation of dental implant placement in the maxillary anterior defect using coDiagnostiX® revealed insufficient bone height and width. (B) Preoperative 3D images. (C) Osteotomy lines and the required bone augmentation were planned. (D) A 3D-printed model was created, and the titanium mesh was shaped to secure space for PCBM grafting.

**Figure 4:**
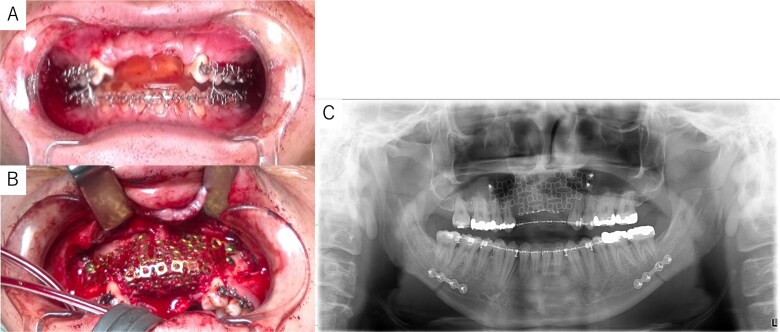
Intraoperative and postoperative images. (A) SSRO surgery was performed with conformity to the orthodontist-prepared final splint. (B) PCBM was grafted into the bone defect and covered with titanium mesh. (C) Postoperative panoramic radiograph showed no abnormalities.

Simulation of dental implant placement in the maxillary anterior region using coDiagnostiX® (Straumann, Basel, Switzerland) revealed insufficient bone volume in both height and width (Fig. 3A). A 3D simulation was performed for bone augmentation in the maxillary anterior region alongside posterior mandibular movement. Mandibular retraction measured 6.2 mm on the right side and 7.0 mm on the left side. DICOM files from dental cone-beam computed tomography were converted to Standard Tesselation Language (STL) data using Volume Extractor® (Volume Extractor 3.0; i-Plants Systems Corporation, Iwate, Japan) (Fig. 3B). Osteotomy lines and the required bone augmentation were established using Geomagic Freeform® (3D Systems, Rock Hill, SC, USA) (Fig. 3C). A 3D model was printed (Straumann® CARES® P20+; Straumann, Basel, Switzerland) from the STL data, and a titanium mesh (Universal Mesh; Stryker Japan, Tokyo, Japan) was shaped to maintain space for PCBM grafting (Fig. 3D). The planned augmentation volume for implant placement was 5.6 mL, with a target bone harvest volume of 9 g, sourced from the right anterior iliac crest. In October 2023, SSRO and bone augmentation were performed simultaneously as planned (Fig. 4A). After harvesting PCBM from the right anterior iliac crest, SSRO was completed. The harvested PCBM was grafted into the bone defect and secured with the pre-bent titanium mesh (Fig. 4B). The wound was sutured, concluding the first operation.

**Figure 5:**
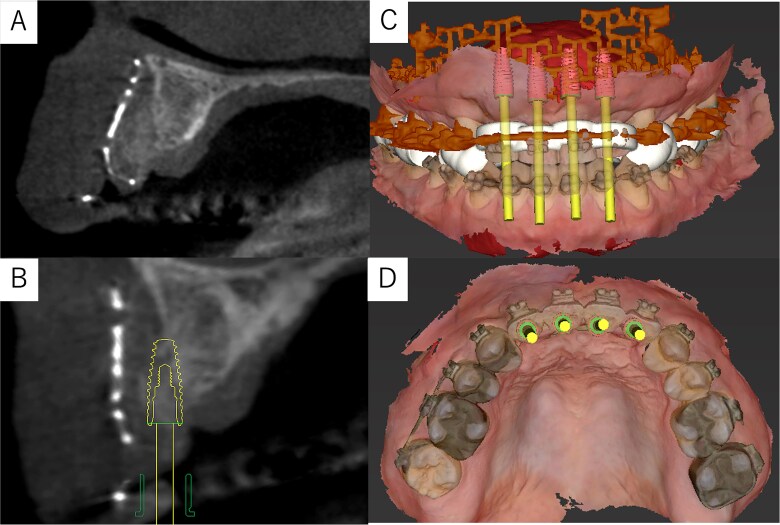
Post-surgery cone-beam computed tomography and implant simulation. (A) Cone-beam computed tomography at 2 months postoperatively confirmed grafted bone stability. (B) Simulation of dental implant placement with a sagittal cross-sectional cone-beam computed tomography image. (C) Frontal view of implant placement. (D) Occlusal view of implant placement.

**Figure 6:**
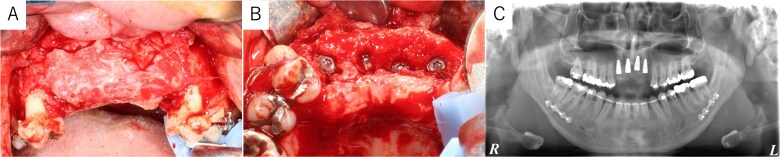
Dental implant placement at 4 months postoperatively. (A) Titanium mesh removal confirmed successful bone augmentation. (B) Dental implants were placed as planned. (C) Postoperative panoramic radiograph confirmed accurate implant placement.

**Figure 7:**
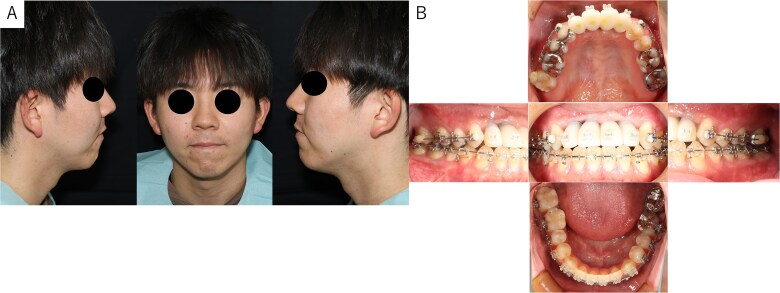
Findings at 1 year postoperatively. (A) Extraoral view showing improvement in skeletal mandibular prognathism. (B) Intraoral view showing the final prosthesis with improved occlusion.

The postoperative panoramic radiograph showed no abnormalities (Fig. 4C). Cone-beam computed tomography images taken 2 months after surgery confirmed the stability of the grafted bone (Fig. 5A). A new simulation for dental implant placement was performed (Fig. 5B–D). At 4 months postoperatively, the titanium mesh was removed, and dental implants were placed. Bone tissue in the defect was confirmed. Implants with a diameter of 4.1 mm and a length of 10 mm (Straumann BLT/RC; Straumann, Basel, Switzerland) were successfully inserted (Fig. 6A–C). Postoperative photographs (Fig. 7) showed improvement in the skeletal mandibular protrusion and a favorable intermaxillary relationship between the upper and lower jaws.

## Discussion

Preoperative computer simulation for corrective surgical procedures has become increasingly common in recent years [6, 7]. In this case, Geomagic Freeform® was used to perform a 3D simulation of bone augmentation and SSRO. Xia *et al*. described a method for simulating orthognathic surgery by creating a bite jig for occlusal configuration, capturing computed tomography images, and superimposing these with DICOM data from a plaster model to create an accurate composite of the dentition and jawbone [8]. More recently, similar software has been developed for simulating orthognathic surgery using this approach [9].

The required PCBM volume was calculated based on the defect region in the jawbone model. Onodera et al. reported that the amount of bone harvested should be twice the predicted volume because the simulated volume is often insufficient during surgery [10]. In this case, the predicted PCBM volume was 5.6 mL, but 10 mL was ultimately required for transplantation—approximately double, consistent with prior findings.

With this approach, it is possible to combine simulated jaw reconstruction with simulated surgical orthodontic treatment. Separating SSRO and jaw reconstruction would typically require three separate surgeries under general anesthesia. Our method reduces the number of surgeries, medical costs, and the patient’s physical burden.

To the best of our knowledge, no similar cases have been reported. This case demonstrates successful esthetic and functional reconstruction of a maxillary anterior bone defect and jaw deformity using preoperative 3D simulation.
